# Chronic Inflammation, Oxidative Stress and Metabolic Plasticity: Three Players Driving the Pro-Tumorigenic Microenvironment in Malignant Mesothelioma

**DOI:** 10.3390/cells12162048

**Published:** 2023-08-11

**Authors:** Irene Fiorilla, Simona Martinotti, Alberto Maria Todesco, Gregorio Bonsignore, Maria Cavaletto, Mauro Patrone, Elia Ranzato, Valentina Audrito

**Affiliations:** 1 Department of Science and Technological Innovation (DISIT), University of Eastern Piedmont, 15121 Alessandria, Italy; irene.fiorilla@uniupo.it (I.F.); simona.martinotti@uniupo.it (S.M.); albertomaria.todesco@uniupo.it (A.M.T.); gregorio.bonsignore@uniupo.it (G.B.); mauro.patrone@uniupo.it (M.P.); elia.ranzato@uniupo.it (E.R.); 2 Department of Integrated Activities Research and Innovation (DAIRI), Public Hospital Azienda Ospedaliera “SS. Antonio e Biagio e Cesare Arrigo”, 15121 Alessandria, Italy; 3 Department for Sustainable Development and Ecological Transition (DISSTE), University of Eastern Piedmont, 13100 Vercelli, Italy; maria.cavaletto@uniupo.it

**Keywords:** mesothelioma, inflammation, tumor microenvironment, oxidative stress, macrophages, DAMP, metabolic adaptation, phenotype plasticity

## Abstract

Malignant pleural mesothelioma (MPM) is a lethal and rare cancer, even if its incidence has continuously increased all over the world. Asbestos exposure leads to the development of mesothelioma through multiple mechanisms, including chronic inflammation, oxidative stress with reactive oxygen species (ROS) generation, and persistent aberrant signaling. Together, these processes, over the years, force normal mesothelial cells’ transformation. Chronic inflammation supported by “frustrated” macrophages exposed to asbestos fibers is also boosted by the release of pro-inflammatory cytokines, chemokines, growth factors, damage-associated molecular proteins (DAMPs), and the generation of ROS. In addition, the hypoxic microenvironment influences MPM and immune cells’ features, leading to a significant rewiring of metabolism and phenotypic plasticity, thereby supporting tumor aggressiveness and modulating infiltrating immune cell responses. This review provides an overview of the complex tumor–host interactions within the MPM tumor microenvironment at different levels, i.e., soluble factors, metabolic crosstalk, and oxidative stress, and explains how these players supporting tumor transformation and progression may become potential and novel therapeutic targets in MPM.

## 1. Introduction

Malignant pleural mesothelioma (MPM) is a rare—even if its incidence all over the world is growing—aggressive, and incurable cancer of the pleural surface [1,2]. The use of surgery in patients with MPM is very limited, the only approved therapy regimen includes pemetrexed plus cisplatin, and there is no standard second-line treatment [3,4]. In recent years, several new therapeutic strategies have been explored, the most promising being immune checkpoint inhibitors (ICIs), blocking the programmed cell death protein 1/programmed cell death1 ligand 1 (PD-1/PD-L1) and cytotoxic T-lymphocyte antigen-4 (CTLA-4), which are currently the standard of care for many advanced solid and hematologic malignancies [5], and anti-angiogenic strategies [6,7,8]. Recently, in the CheckMate 743 trial, the combination of nivolumab and ipilimumab as a first-line treatment showed a significant survival benefit versus standard chemotherapy, mainly in the non-epithelioid subtype, suggesting a potential use as a first line in the future [9]. As a second-line treatment [3,10], preliminary uncontrolled trials of ICIs have suggested encouraging activity, but with contrasting results in terms of survival improvement in different clinical studies [11,12]. Many efforts are ongoing to find predictive factors that can help to select the suitable candidate patients for immunotherapy [2,13]. A better understanding of tumor heterogeneity, as well as the identification of novel biomarkers able to predict prognoses and responses to therapy, are thus of paramount importance for the development of novel therapeutic strategies leading to patient cure.

Asbestos exposure is the most important risk factor in the formation of MPM, with a latency period of up to 40 years after exposure, even if the exact molecular mechanisms that explain its carcinogenicity are complex and not fully known [14,15]. MPM arises on the serosal surfaces of the mesothelial membrane of the pleura. It is histologically subdivided into three subtypes: epithelioid, biphasic/mixed type, and sarcomatoid. The epithelial subtype is the most diffuse (50% of cases) and is associated with the best overall prognosis. On the contrary, the sarcomatoid subtype (25% of cases) is associated with a poor prognosis [16].

Many genetic factors can influence MPM carcinogenesis and outcomes, such as mutations in tumor suppressor genes such as cyclin dependent kinase inhibitor 2A (*CDKN2A*), BRCA1 associated protein 1 (*BAP1*), neurofibromatosis 2 (*NF2*), and *TP53* [8,17]. However, chronic inflammation is the main factor underlying the pathogenesis of MPM induced by asbestos, leading to malignant mesothelial cell transformation. Mechanistically, longer fibers cannot be effectively phagocytized by macrophages. The resulting “frustrated phagocytosis” promotes the inflammatory cells surrounding the fibers to release free radicals such as reactive oxygen species (ROS) [18,19]. Stressed macrophages and mesothelial cells undergo cell necrosis, releasing the damage-associated molecular protein (DAMP) high mobility group box 1 (HMGB1) [20]. In turn, HMGB1 and pro-inflammatory cytokines promote the recruitment of macrophages and, together with ROS, sustain the chronic inflammatory process and DNA damage leading to mesothelial cell transformation over the years [1,21].

This review will provide the current understanding of the complex tumor–host interactions within the MPM tumor microenvironment at different levels, i.e., soluble factors, metabolic crosstalk, and oxidative stress, and clarify how these players may become potential and novel therapeutic targets in MPM.

## 2. Inflammation and Mesothelioma Pathogenesis

A chronic inflammatory state is the essential element characterizing the pathogenesis of MPM. The picture is exceptionally complex, with numerous cellular and environmental interactions building a unique tumor microenvironment (TME) supporting malignant mesothelial cell transformation and proliferation.

The onset of mesothelioma is associated with exposure to asbestos, and consequently, the activation of immune system cells in the surrounding tissue/TME, leading to oxidative stress with ROS release and the secretion of pro-inflammatory factors amplifying the process of inflammation [22].

Consistently, the over-activation of the immune system is correlated with worse patient outcomes and poor responses to treatment. Furthermore, another important kind of inflammation is therapy-induced inflammation, which progresses in response to various anti-neoplasm therapies, including radiotherapy, chemotherapy, and, recently, immunotherapies or biologic therapies, leading to a huge infiltration of immune cells [23].

Mechanistically, during mesothelioma development, when mesothelial cells experience asbestos, fibers secrete several macrophage-attractant factors, including interleukin (IL)-8, IL-6, C-C motif chemokine ligand 2 (CCL2 or also called monocyte chemoattractant protein-1, MCP-1), granulocyte colony-stimulating factor (G-CSF), granulocyte/macrophage colony-stimulating factor (GM-CFS), and macrophage inflammatory protein-1 (MIP)-1alpha, which start the inflammatory cascade, and, in turn, recruit other monocytes at the tumor site [24,25,26]. The activation of the CCL2/CCR2 axis, which mediates the crosstalk between tumor-associated macrophages (TAMs) and tumor cells, is associated with angiogenesis, metastases, immunosuppression, and cancer progression [27,28]. A huge number of infiltrating M2-like macrophages (CD163+; CD206+; and IL-4Rα+) have been found in MPM [24,29]. Physiologically, macrophages act via phagocytosis to destroy foreign antigens [30]. However, in MPM, the defective phagocytosis of asbestos fibers by macrophages contributes to the creation of a TME that sustains the survival of mutated mesothelial cells, leading to carcinogenesis. These “frustrated macrophages” release oxidative mediators and pro-inflammatory cytokines that sustain an inflamed environment and trigger signaling pathways in transformed mesothelial cells that help them to survive, despite the asbestos-related damage [31], as detailed in the next sections. Additionally, TAMs–MPM crosstalk underlies the acquisition of the immunosuppressive and chemoresistant phenotype [32]. Targeting TAMs alone or in combination with other therapies may be a promising therapeutic approach for cancer therapy, including in MPM [33].

## 3. Mesothelioma Microenvironment: Tumor–Host Crosstalk Driving Chronic Inflammation

### 3.1. Secretome/Soluble Factors

**Figure 1:**
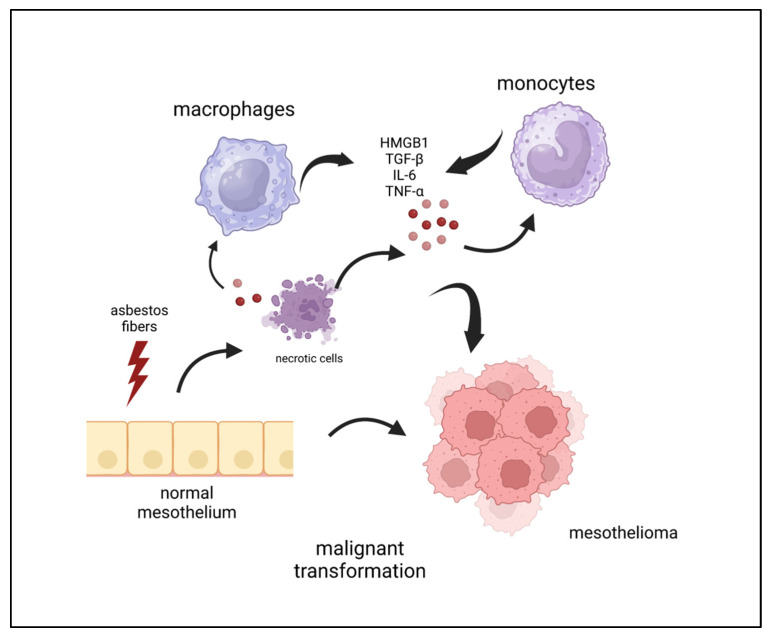
Cellular and soluble factors involved in asbestos-induced mesothelial cell transformation. Upon exposure to asbestos, necrotic cells and monocytes/macrophages release/secrete soluble factors creating an inflamed environment able to support the onset and the progression of mesothelioma. See main text for abbreviations. Created with BioRender.com.

Several secreted factors create an inflamed milieu, supporting the pathogenesis and the progression of MPM. Herein, we describe the principal cytokines/factors involved in MPM chronic inflammation, as summarized in Figure 1.

#### 3.1.1. Transforming Growth Factor-β (TGF-β)

The transforming growth factor-β (TGF-β) family is a group of polypeptides able to control growth, morphogenesis, and cellular differentiation [34]. In the early stages of tumorigenesis, TGF-β can act as a tumor suppressor; however, during cancer progression, TGF-β can activate cell growth, cancer progression, and dissemination through the promotion of epithelial-to-mesenchymal transition (EMT), angiogenesis, and escape from immune system [34].

In mesothelioma, some TGF-β family members, such as gremlin-1 [35], activin A [36], and TGF-β itself [37], have been recognized as being involved in cancer development. Moreover, most human mesothelioma cell lines secrete important amounts of TGF-β, and mesothelioma tissue sections show TGF-β positivity [38]. Notably, TGF-β pathway blocking can lead to a substantial inhibition of the in vivo growth of mesothelioma [39]. Furthermore, a study that explored 74 mesothelioma tumors using a genomic analysis identified TGF-β1 mRNA expression as being correlated with the worst patient prognoses [40].

According to these data on the role of TGF-β, fresolimumab, a human anti-transforming growth factor-beta monoclonal antibody, has been tested in patients with advanced mesothelioma [41]. In this study, no evident radiographic responses were observed, and the median progression time was short (1.4 months), with 10 patients progressing after only two cycles of treatment.

However, new findings [42] on the suitability of TGF-β level, using pleural effusions as a reliable diagnostic biomarker, may pave the way to further understanding TGF-β’s role in the pleural space.

#### 3.1.2. IL-6 in Mesothelioma

IL-6 is a pleiotropic cytokine released by a plethora of cells, such as B cells, T cells, and macrophages, but also fibroblast, endothelial cells, and epidermal keratinocytes [43]. IL-6 is a soluble mediator exerting a pleiotropic effect on the immune response, inflammation, and hematopoiesis [44].

Mesothelioma cells produce IL-6 [45,46], and an elevation of the circulating level of IL-6 has been already described in mesothelioma patients, with this increase being associated with a negative clinical condition. High levels of IL-6 have been also revealed in patients’ pleural fluids [47].

Monoclonal antibodies raised against IL-6 are able to block the clinical symptoms in in vivo mesothelioma mouse models [45].

However, there are conflicting results concerning the role of IL-6 as an autocrine growth factor for mesothelioma. Some authors [45,46] have defined that, in mesothelioma cells, IL-6 is not able to induce tumor growth and anti-IL-6 therapy does not induce significant results in terms of reducing cancer growth. Despite these observations, other authors [48] have concluded that IL-6 can play a putative role as an autocrine molecule via a signal transducer activator of the transcription 3 (STAT3) pathway.

Thus, despite other malignancies where there is a clear correlation between IL-6 concentrations and poor prognostic factors [49], such a role in mesothelioma has not been defined. However, IL-6 is worthy of investigation, because it is emerging as a possible resistance mediator to classic cytotoxicity agents [50].

#### 3.1.3. Tumor Necrosis Factor-α (TNF-α) and Asbestos Exposure

TNF-α is a cytokine (17KDa, 157 amino acids) mainly released by activated macrophages. TNF-α receptors are expressed in macrophages and other organs. TNF-α is a pro-inflammatory molecule and one of the main inducers of nuclear factor kappa B (NF-κB). The activation of NF-κB stimulates cell growth and inhibits cell death, supporting cancer development [51].

TNF-α signaling plays a pivotal role in mediating the responses of mesothelial cells to asbestos fibers. Asbestos fiber protracted inhalation can lead to chronic inflammation within the pleura, inducing carcinogenic progression. Asbestos fibers damage the integrity of the mesothelial tissue, provoking a local inflammation process with the overproduction of free radicals and release of pro-inflammatory cytokines [52]. Therefore, TNF-α and its downstream NF-κB signaling pathway act important roles for the beginning of the inflammatory process. After mechanical damage, the mesothelial tissue can also secrete HMGB1, inducing the recruitment of macrophages and release of TNF-α [53].

TNF-α exerts its role acting on its receptor, TNF-R1, directly expressed on mesothelial cells’ plasma membrane. This interaction induces another autocrine release of TNF-α by mesothelial cells, the activation of NF-κB signaling, and the final result of skipping the apoptotic process of the injured mesothelial cells [54].

#### 3.1.4. DAMP and Mesothelioma

Stressed or injured cells release a huge number of mediators, generally called DAMPs, that strongly generate sterile inflammation. DAMPs comprise a large variety of chemically different molecules, such as S100 proteins, heat shock proteins, hyaluronan, ATP, calreticulin, and HMGB1. These molecules are retained by the cells in the healthy state and released only after cell death or stress [55].

Initially, DAMPs were considered to be released only from necrotic cells, while new findings support the evidence that immunogenic cell death and necroptosis can trigger DAMPs’ release from cytosol into the extracellular space [56].

#### 3.1.5. HMGB1: What It Is and How It Can Play a Role in MPM

The HMGB1 protein is a member of the highly conserved non-histone DNA-binding protein family, so named for its characteristic rapid mobility in polyacrylamide gel electrophoresis [57]. In the nucleus, HMGB1, acting as a chaperonin, participates in DNA repair and transcription, interacting with transcription factors such as p53. In the nucleus, HMGB1 also improves telomerase activity [58].

Furthermore, HMGB1 can be released into the extracellular space via two mechanisms: passive release and active secretion. HMGB1 is passively released from necrotic or damaged cells, causing a direct inflammatory cascade via pro-inflammatory molecules such as TNF-α. HMGB1 is actively secreted by endothelial cells, immune cells, neurons, platelets, astrocytes, and tumor cells during stress conditions, or secondary to other DAMP danger signals as support [58].

Cytoplasmic HMGB1 is involved in immune responses by inhibiting apoptosis, increasing autophagy, and regulating mitochondrial functions [59]. HMGB1 also plays a role in triggering inflammation, but it would also seem to be involved in innate and adaptive responses and the repair of tissue damage [58].

HMGB1 is an intriguing molecule and appears to play an important role in tumor biology, inflammation, and cancer progression. HMGB1 overexpression has been observed in several neoplasms, such as breast cancer, pancreatic cancer, melanoma, and mesothelioma [60]. HMGB1, released into the extracellular space, operates as a signaling molecule interacting with different surface receptors, including the receptor for advanced glycation end-products (RAGE), C-X-C motif chemokine receptor 4 (CXCR4), mucin domain-containing protein 3 (TIM3), T cell immunoglobulin, and Toll-like receptors (TLRs). The engagement of these receptors leads to cell proliferation, escape from apoptosis, enhancement of the process of invasion and angiogenesis, metastasis, increased inflammation, and immune activation.

HMGB1 also appears to be correlated with malignant mesothelioma [61,62]. High levels in the blood of both subjects exposed to asbestos and mesothelioma patients have been demonstrated [62].

A proposed model of the pro-tumor or antitumor activities of HMGB1 has been elucidated. Once inhaled, asbestos fibers are quickly recognized by immune cells and in particular by macrophages, and may break down into shorter fibers and particles in the lungs, also forming asbestos bodies [53]. Similarly, asbestos fibers can promote mesothelial cells’ inflammation and programmed necrosis. Then, HMGB1 signals cellular injury in response to damage and inflammation. Upon asbestos exposure, HMGB1, as a pro-inflammatory cytokine, stimulates macrophages to release TNF-α, protecting cells from asbestos-induced cell death and triggering a continuous inflammatory cascade. Combined with previous findings, this evidence advises that HMGB1 could be considered as a key inflammatory intermediary involved in the mechanism of carcinogenesis underlying mesothelioma development.

#### 3.1.6. Peripheral Blood Markers

Peripheral blood tests before starting a treatment may reveal a patient’s inflammatory status with cancer, including mesothelioma. Moreover, chronic inflammation is deeply involved in the onset of mesothelioma and some scores related to inflammation could be useful as potential prognostic markers, such as lymphocyte-to-monocyte ratio (LMR), neutrophil-to-lymphocyte ratio (NLR), and platelet-to-lymphocyte ratio (PLR).

The NLR has been described as a poor prognostic marker in neoplastic patients. Nevertheless, its prognostic role in mesothelioma has not been completely defined [2]. However, recent studies have proposed that NLR elevation could be a potential prognostic factor for mesothelioma patients [63].

In contrast, the LMR has been recognized as an independent prognostic marker for survival in patients, and the LMR is superior to other inflammation-based prognostic scores [64]. PLR was identified as an independent prognostic factor in mesothelioma patients, representing a new scoring system for predicting disease outcomes [65].

### 3.2. Oxidative Stress and ROS

**Figure 2:**
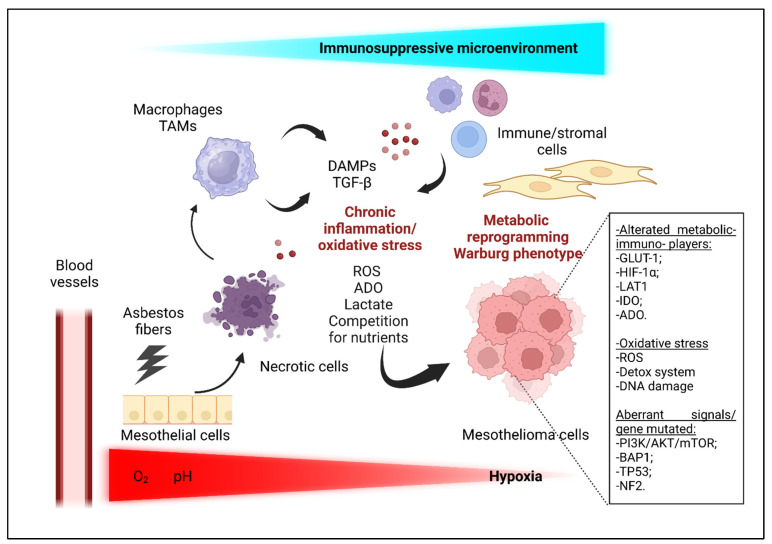
Key players within the MPM microenvironment. The complex landscape of MPM-TME is characterized by the interconnection between cancer and stromal/immune cells to create a permissive and pro-tumorigenic environment. Hypoxic conditions and oxidative stress, chronic inflammation, and soluble factors trigger metabolic reprogramming. Moreover, also aberrant signaling and DNA damage pathways, as well as mutations in specific genes such as *BAP1*, contribute to a switch toward glycolytic metabolism (Warburg effect) in tumor and in immune cells, promoting MPM cell growth and progression. Lactate and immunosuppressive molecules, including IDO and ADO excreted in the microenvironment, lead to MPM cells’ adaptation, survival, metastatization, and escape from immune control. See main text for abbreviations. Created with BioRender.com.

Chronic oxidative stress, with a huge release of ROS by frustrated macrophages and tumor cells, is a second hallmark of mesothelioma carcinogenesis following asbestos fibers exposure [15,66] (Figure 2).

Oxidative stress is defined as an imbalance between the production of free radicals and oxidants or ROS (i.e., superoxide, hydrogen peroxide, and hydroxyl radicals), and the ability of antioxidant biological systems to quickly detoxify reactive metabolites or repair the resulting injury [67].

ROS are highly chemically reactive molecules, derived from the deficient reduction in molecular oxygen (O_2_) and playing a critical role as signaling molecules in tumorigenesis [68], including asbestos-mediated carcinogenesis [15,66,69]. ROS, which are produced by cancer cells and non-cancer cells within the microenvironment, are essential elements connecting metabolic reprogramming/oxidative stress/inflamed environment, all of which are key aspects of cancer progression [70]. Most endogenous ROS generated in cells are derived from metabolic reactions happening within the mitochondria or peroxisomes, while in tumor cells, ROS are primarily produced by the mitochondria. Mitochondria generate superoxide (O_2_·) through the mitochondrial electron transport chain (ETC) [71,72]. Moreover, ROS can be generated by enzymatic mechanisms involving endothelial nitric oxide synthase (eNOS), nicotinamide adenine dinucleotide phosphate (NADPH) oxidases (NOXs), arachidonic acid metabolism, lipoxygenase, xanthine oxidase, cyclooxygenase, and enzymes belonging to the cytochrome P450-dependent redox machinery [72]. Therefore, the coordination of ROS/redox homeostasis is pivotal for regulating physiological and pathological biological functions, including cell growth and survival, senescence, and aging [67,73]. The elevated ROS levels in tumor cells are balanced by the increase in the antioxidant enzymatic and non-enzymatic machinery and pathways that scavenge ROS [74]. To do this, cells exploit a spectrum of antioxidants, and among those synthetized by tumor cells themselves, we can find non-catalytic small molecules such as α-lipoic acid, bilirubin, uric acid, melanin, melatonin, and glutathione (GSH). In addition, some antioxidant molecules are exogenously derived, including the non-catalytic small molecules β-carotene vitamin E and C, and plant polyphenols, which can participate in the ROS scavenging process [67,74]. GSH is often overexpressed by cancer cells to overcome ROS accumulation [67,75] Cells also have catalytic antioxidants able to scavenge the superoxide anion O_2_·, converted into hydrogen peroxide (H_2_O_2_) by the cytosolic copper/zinc superoxide dismutases (SOD) 1 and 2, localized in the cytosol and mitochondria, respectively [68]. H_2_O_2_ can then be converted by catalases (CATs), glutathione peroxidases (GPXs), or thioredoxin peroxidase (TrxP) into H_2_O and O_2_ [76].

The generation of ROS is increased in cancer cells compared to normal cells due to elevated energetic metabolic rates, gene mutations, and microenvironmental conditions such as hypoxia [68]. ROS play an essential role in carcinogenesis, affecting multiple biological processes, including genomic instability, cell growth, angiogenesis, inflammation, metabolic reprogramming, metastatization, and resistance to apoptosis [77]. ROS contribute to tumor progression, also modulating signaling cascades such as mitogen-activated protein kinase (MAPK), phosphoinositide 3-kinase (PI3K), and NF-κB. On the other hand, elevated ROS levels lead to macromolecular damage by oxidizing nucleic acids, lipids, and proteins, which can promote cell death [77].

In MPM pathogenesis, the retention of long asbestos fibers in the respiratory tract, coupled with the defeated phagocytosis of pleural macrophages that try to ingest them, increases oxidative stress with ROS generation and forces continuous inflammation, which may promote mesothelial cell transformation [78,79]. Asbestos fibers can generate ROS via two different mechanisms: the first one involves the iron (Fe) content of the fibers, increasing hydroxyl radical (HO·) production through iron-catalyzed reactions; and the second mechanism implies the release of ROS upon the over-activation of inflammatory immune cells [66,78].

As mentioned before, ROS accumulation is followed by the activation of the cellular antioxidant defense. In response to high ROS levels and DNA damage, mesothelioma cells overexpress nuclear factor erythroid 2-related factor 2 (Nrf2), redox effector factor 1 (Ref-1), and forkhead box protein M1 (FOXM1) involved in the antioxidant pathways to reduce oxidative stress. Schiavello et al. demonstrated that these redox-sensitive transcription factors that can counteract MPM chronic oxidative stress can therefore be considered as predictive biomarkers of MPM and potential pharmacological targets in the treatment of MPM [80].

An interesting interplay between oxidative stress and pro-inflammatory cytokines is represented by the ROS–TGF-β axis. As previously mentioned in this review, TGF-β itself and its family members have been recognized to be involved in cancer development [81], and TGF-β possesses a pro-oncogenic role in MPM connected to immune suppression [82,83]. It has been described that extracellular ROS regulate the TGF-β axis [66,84,85]. Pociask et al. showed that ROS released by asbestos-associated iron promoted the oxidation of latent-associated peptide (LAP) and the activation of TGF-β1. The supplementation of culture media with antioxidants before asbestos exposure counteracted this effect, clearly showing the impact of ROS on TGF-β1 activation [86]. TGF-β1 plays a crucial role in promoting EMT, a key process involved in modulating alveolar epithelial cell plasticity [87,88]. ROS can sustain EMT via TGF-β1 [89]. Recently, Turini et al. demonstrated that human mesothelial cells (MeT-5A) exposed to chrysotile asbestos activate an EMT program, up-regulating mesenchymal markers including vimentin, fibronectin, and α-SMA, while down-regulating epithelial molecules such as E-cadherin, β-catenin, and occluding. This phenotypic plasticity is mediated by the over-expression of TGF-β1 [90]. In turn, TGF-β can promote ROS production via the ETC [91] and down-regulate the expression of antioxidant enzymes [84], suggesting a circuit of positive mutual regulation. This axis could be pharmacologically targeted, opening up a possibility for novel combination therapy [92].

#### Iron and ROS

The essential trace element Fe plays a multifaceted and complex role in tumor biology [93]. Several studies have shown that iron metabolism is involved in tumor occurrence and development [94]. In addition, iron is involved in regulating the cell death process, which is being exploited for the design of novel potential anti-tumor therapies. Therefore, iron shows a paradoxical role in tumors: iron is required by the cancer cell to sustain its growth and proliferation [93,95]; on the other hand, iron overload induces oxidative stress, producing many hydroxyl radicals, which can lead to lipid peroxidation. If the cellular antioxidant systems are overwhelmed, iron can trigger ferroptosis, a non-apoptotic, iron-dependent cell death, driven by an overload of lipid peroxides on the cellular membrane. Ferroptosis can be considered as a natural barrier to cancer development, triggered by the activities of several tumor suppressors, including p53 and BAP1, also altered in mesothelioma [96]. On the contrary, ferroptosis evasion and resistance may contribute to tumor initiation and progression [97].

In addition, cancer cells themselves can modulate iron homeostasis, rewiring their metabolism. Hypoxic cancer cells need higher concentrations of iron to sustain their aggressiveness and redox balance [96]. Iron uptake is elevated and/or iron efflux is decreased, influenced by indirect or direct regulatory pathways [96,98].

Some studies have highlighted a role for the iron tumorigenic process induced by asbestos [18,99]. The presence of asbestos fibers in tissues provides a surface for iron-rich macromolecular aggregates (asbestos bodies), supporting the creation of chronic inflammatory conditions and oxidative tissue damage, even if the molecular mechanism remains elusive [18,19]. A de-regulation of iron-heme machinery and transport (i.e., the proteins involved in iron absorption, iron storage, and ferritinophagy, as well as heme synthesis, heme degradation, and heme export) could also be linked with MPM carcinogenesis. For example, the core subunit of iron-binding protein ferritin, the ferritin heavy chain (FHC), in mesothelial and mesothelioma cells, in the presence of asbestos or oxidative stress, functions as an anti-apoptotic protein [100]. In particular, Aung et al. found that asbestos exposure induces FHC protein expression in human mesothelial cells. Mesothelial cells that stably express the FHC have a smaller amount of H_2_O_2_ upon asbestos exposure. Moreover, mesothelial cells expressing higher levels of FHC are more resistant to apoptosis induced by H_2_O_2_. The expression of FHC is correlated with a reduced production of H_2_O_2_ [100]. The other subunit of ferritin, the ferritin light chain (FTL), is able to affect p27, p21, cyclin-dependent kinase 2 (CDK2), and retinoblastoma protein (pRB) expression, increasing the proliferative capacity of malignant mesothelioma cells and promoting the cell cycle [101]. Finally, it has been reported that iron-catalyzed ROS production and impaired ferroptosis may be involved in asbestos-related carcinogenesis [102]. Future studies will be essential for defining the molecular mechanisms that regulate iron/ROS/oxidative stress to identify novel vulnerabilities and potential drugs for MPM.

### 3.3. Metabolic Crosstalk and Reprogramming

In normal cells, most energy is derived via oxidative respiration (i.e., the tricarboxylic acid (TCA) cycle and oxidative phosphorylation (OXPHOS)). A hallmark of cancer is an alteration in the metabolic pathways, including glycolysis and the TCA cycle coupled with OXPHOS [103,104]. Usually, cancer cells increase the rate of glycolysis, leading to a massive conversion of the pyruvate in lactate, a sort of fermentation, but in the presence of oxygen [105]. This metabolic pathway, called the “Warburg effect” or aerobic glycolysis, protects cancer cells from hypoxic conditions and offers an abundant source of precursors for the synthesis of nucleic acids, phospholipids, and fatty acids, which is necessary for tumor cell proliferation [106,107]. Furthermore, lactate, released into the extracellular space, forces tumor growth and metastatization and sustains tumor immune-evasion mechanisms [108,109]. Adaptation to hypoxia via the Warburg phenotype and the selection of tumor clones able to survive in acidosis conditions leads to a powerful growth advantage for malignant cells [110]. In the tumor microenvironment, activated immune cells (T cells, dendritic cells, and macrophages) also undergo metabolic rewiring, switching between aerobic glycolysis and OXPHOS, depending on the microenvironmental conditions and state of activation [107,111,112].

Mesothelioma cells show a metabolic plasticity [66] (Figure 2). MPM lesions are commonly highly glycolytic, as we describe in the following paragraphs. Several pathways, including those controlled by MYC, PI3K–AKT, hypoxia-inducible factor 1-alpha (HIF-1α), AMP activated protein kinase (AMPK), and p53, regulate the metabolic switch toward aerobic glycolysis; in addition, a loss of function mutations in the *BAP1* gene alter the mitochondrial metabolism, supporting the metabolic adaptation of MPM cells [66] (Figure 2).

#### 3.3.1. Hypoxic Conditions and Nutrients Competition

Hypoxia is one of the essential hallmarks of the mesothelioma metabolome [22,113] (Figure 2). In human MPM, fluctuating oxygen levels promote tumor proliferation, tumor–stromal interactions, angiogenesis, stemness, EMT, phenotype plasticity, and resistance to chemotherapy [113]. In addition, hypoxia forces the metabolic reprogramming of cancers, increasing glucose uptake and the conversion of pyruvate into lactate. In vivo, fluoro-2-deoxy-D-glucose (F-FDG) uptake in pleural mesothelioma shows an elevated correlation with glucose transporter 1 (GLUT-1), HIF-1α, vascular endothelial growth factor (VEGF), CD44, and PI3K/mTOR upregulation [113,114,115,116]. It was demonstrated by Minato et al. that mesothelioma cells can upregulate GLUT-1 to uptake glucose more efficiently. Increased levels of GLUT-1 are often correlated with poorer prognoses [117,118]. In the mesothelioma microenvironment, the availability of glucose is critical, and competition for this molecule impacts on T-cell function and the recruitment of immune infiltrating cells, promoting angiogenesis [119,120,121,122]. In summary, hypoxia raises the acquisition of aggressive phenotypes in human malignant mesothelioma, also supporting metabolic plasticity [113].

Competition for nutrients, not only glucose but also amino acids, is critical within the mesothelioma microenvironment (Figure 2). It is known that mesothelioma cells increase the expression of L-type Amino acid Transporter 1 (LAT1), depriving T-cells of amino acids, such as arginine and tryptophan, which are essential for T-cell function and proliferation [121]. Mesothelioma cells could also inhibit T-cell glycolysis and functions, increasing the levels of indoleamine-pyrrole 2,3-dioxygenase (IDO), which degrades tryptophan into kynurenine, a well-known immunosuppression factor [121,123,124]. In conclusion, the mesothelioma metabolome and secretome equally recruit and reprogram infiltrating immune cells [122].

#### 3.3.2. Adenosine Pathway

Adenosine (ADO) plays important roles in building an immunosuppressive TME contributing to cancer progression [125]. The extracellular (e) levels of ADO in tumors are more elevated than those in normal tissues [125,126]. eADO concentrations can be raised in response to metabolic changes. In conditions of low oxygen or nutrients, we observe an inadequate ATP biosynthesis in cells, leading to a reduction in the ATP/adenosine ratio. Hypoxic conditions lead to a strong accumulation of eADO in tumors, also regulating the expression of ADO production machinery, including CD39 and CD73 ectonucleotides [127,128]. While under normal conditions the Adenosinergic Pathway (AP) is fine-tuned and controlled, in cancer the signaling is altered, promoting malignant cell transformation. AP in cancer cells by increasing the secretion of cytokines and immune modulatory factors, including IL6, IL10, TGFβ, and VEGF, and activating survival/signaling pathways such as PI3K-Akt-mTOR, sustains immunosuppression and promotes tumor progression, metastasis, and therapy resistance [26,129,130].

ADO signaling has been slightly studied in MPM, however, some evidence has supported a functional role of ADO in the MPM immunosuppressive microenvironment (Figure 2). As observed in other tumors [131], the ADO signaling pathways may regulate MPM cells–TAMs interaction, inducing TAMs’ proliferation and the release of pro-tumoral cytokines [129]. Al-Taei et al. showed CD73 expression on TAMs in pleural effusion derived from MPM patients. This result supports the hypothesis that the TAMs–ADO axis is involved in creating immunosuppressive conditions within the TME of mesothelioma [132]. Clayton et al. reported the expression of CD39, CD73, and eADO in MPM-derived exosome from pleural effusion, contributing to the inhibition of T cell functions [133]. Nakajima et al. showed that adenosine deaminase (ADA), an enzyme that catalyzes the deamination of adenosine to inosine and deoxyadenosine to deoxyinosine, is increased in MPM pleural effusions compared to benign diseases, suggesting the existence of an endogenous mechanism able to switch-off ADO signaling [134].

Despite very little information about the role of the AP/ADO axis in MPM being present, these data incentivize researchers to start novel studies to investigate the functional role of this network in MPM, with an eye on its translational and therapeutic potential [26].

#### 3.3.3. BAP1 and Metabolic Reprogramming

The most representative type of mutation in tumor suppressor genes frequently mutated in MPM is a loss of function mutation. Since the finding that germline-inactivating *BAP1* mutations predispose MPM [135], increasing evidence has confirmed this discovery, showing autosomal dominant germline mutations in the *BAP1* gene in almost all MPM patients 50 years old or younger with a family history of mesothelioma and/or uveal melanoma (UVM) or clear cell renal cell carcinoma (ccRCC) [136]. The cooperation between *BAP1* mutations and carcinogens remains not fully defined. In vitro and in vivo studies have indicated that a combination of mutations in *BAP1* and other tumor suppressor genes, as well as in genes involved in DNA repair, increases sensitivity to asbestos, ionizing radiation, and ultraviolet light. *BAP1* mutations simultaneously impair (1) transcription and gene expression, (2) the mechanisms regulating cell death and DNA repair, and (3) metabolic pathways, increasing cancer risk [136,137,138] (Figure 2). However, some malignancies in germline *BAP1* mutation carriers, especially mesotheliomas, are much less aggressive, even if the exact mechanism is not completely understood [139].

BAP1 in involved in the modulation of the cellular metabolism. A reduction in BAP1 protein levels supports the reprogramming of cancer cell metabolism, with a switch from TCA/OXPHOS to aerobic glycolysis [140]. Increased aerobic glycolysis is frequently observed in BAP1+/− cells [141], also analyzing the plasmatic metabolic profiles of individuals carrying different germline *BAP1* mutations in comparison to *BAP1* wild-type (BAP1WT) controls derived from two families [140]. Consistently, in cell culture media from MPM primary cells derived from patients carrying germline *BAP1* mutations, an increased level of extracellular lactate was observed [140]. Lactate is known to promote tumor growth [142], angiogenesis [143], and polarize macrophages to express a pro-tumor M2 phenotype [144]. As reported by Bononi et al., BAP1 regulates ER-to-mitochondria calcium (Ca^2+^) release; thus, mutations in *BAP1* lead to the intracellular Ca2+ reduction required for the regulation of OXPHOS enzymes, resulting in an impairment of mitochondrial respiration and a switch toward glycolysis [139,140]. In addition, Baughman et al., using an inducible knockout mouse model of Bap1, demonstrated modified metabolic pathways due to Bap1 deletion, including increased cholesterol biosynthetic machinery, paralleled by a reduced expression of lipid homeostasis and gluconeogenic proteins, as well as a reduced expression of mitochondrial proteins in the liver and pancreas [145]. BAP1 regulates several activities by forming multi-protein complexes [146], stabilizing the master regulator of mitochondrial biogenesis peroxisome proliferator-activated receptor-γ coactivator-1α (PGC-1α) and promoting gluconeogenesis [147]. Therefore, BAP1 alterations impact on metabolic homeostasis, favoring tumor development. Future studies on understanding the key role of BAP1 in tumors, especially in mesothelioma, could lead to the development of novel therapies with substantial clinical improvements.

## 4. Conclusions and Perspectives

MPM is a highly aggressive, incurable cancer linked to asbestos exposure. To date, despite the novel and promising strategies targeting its TME, such as immunotherapy and the combination of immuno/chemotherapy, included in ongoing clinical trials on MPM, as recently reviewed in [8,26,148,149,150], the efficacy of these therapeutic approaches remains to be confirmed. For these reasons, it is strictly necessary to identify novel and more effective combination therapies that may target multiple vital cellular checkpoints, as well as determine predictive markers of responsiveness to therapy. Moreover, MPM is a challenging malignancy to manage, as it is often difficult to diagnose. Currently, a diagnosis of MPM is made often in the late stages of the disease, principally by pathologists and based on biopsied or resected tissue specimens. This evidence highlights the importance of discovering novel biomarkers, both tumoral and blood-based.

MPM shows a unique architecture of the TME, where, as described in this review, complex and multiple interactions between mesothelioma, stromal, and immune cells contribute to the creation of pro-tumoral and immunosuppressive conditions that favor the progression of the disease. Moreover, not only cell–cell interactions, but also soluble factors, including cytokines, DAMPs, metabolites, and ROS secreted and/or released within the TME foster chronic inflammation/oxidative stress, impacting on immune-evasion mechanisms and response to therapy. Lastly, the metabolic rewiring occurring in tumor and immune cells and the metabolic crosstalk between cells generate conditions that support malignant cell aggressiveness and drug resistance.

Therefore, recent advances have shed light on the pathogenesis and tumor microenvironment components of MPM, despite the significant intra-tumoral and inter-tumoral heterogeneity between patients. However, we hope that a more comprehensive description of these processes and mechanisms in the future could assist in finding novel tumor vulnerabilities that can be more effectively targeted to cure this aggressive disease, or at least increase the overall survival of MPM patients.

## Data Availability

Not applicable.
